# Supplementary figures and images for: A scoping study of the whole-cell imaging literature as a foundation for the emerging field of cell anatomy
Source: BMC Biol. 2026 Feb 27;24:91. doi: 10.1186/s12915-026-02556-0 (PMC13049767; doi:10.1186/s12915-026-02556-0)

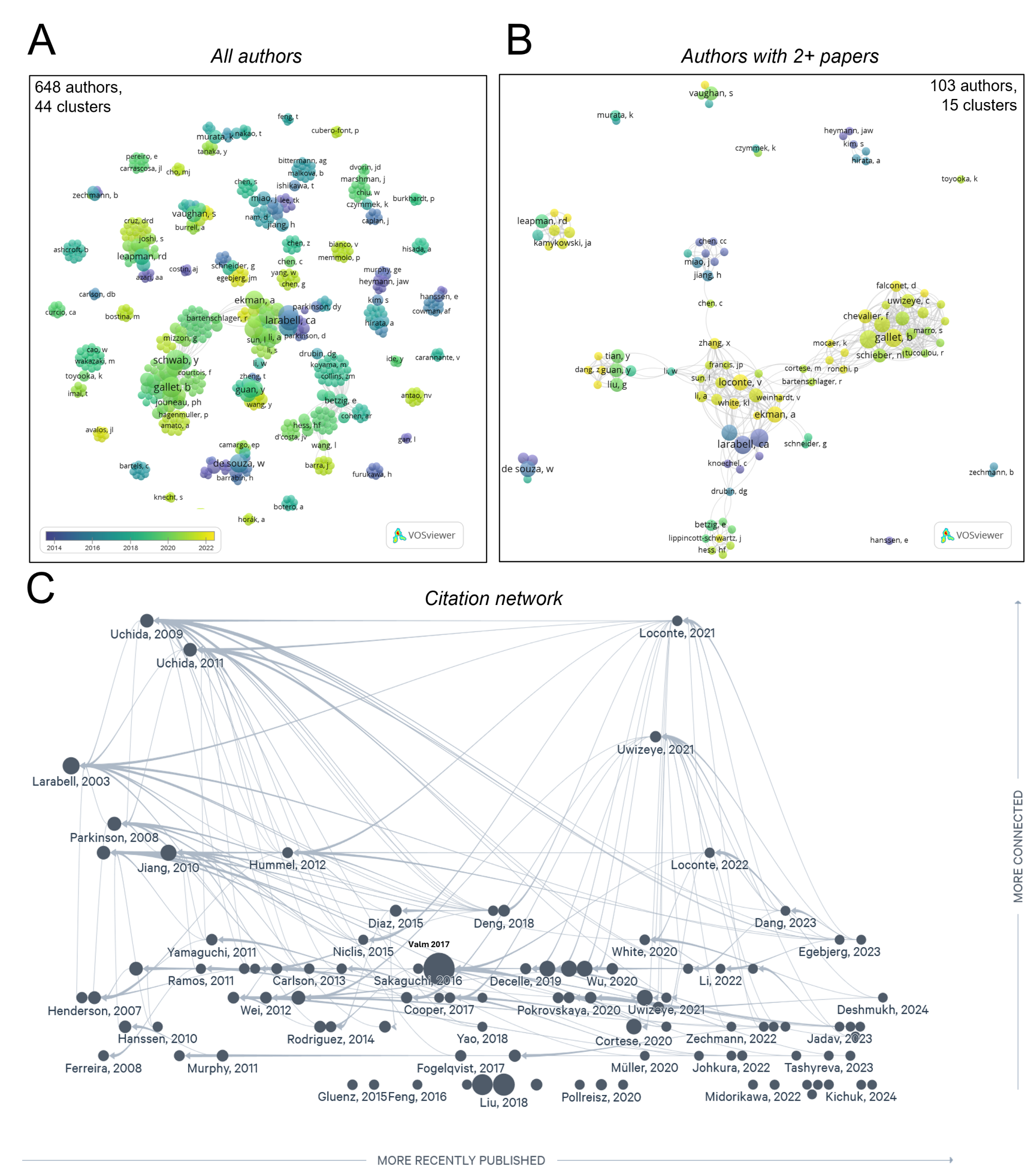

Supplement: Supplementary file 6 — Additional file 6. Fig. S1 Bibliometric network analysis. A–B) Co-authorship network consisting of authors with A) any number of publications and B) those with at least two publications represented in the corpus. Color gradient represents average publication year per author and bubble size indicates relative number of articles per author. Visualizations created in VOSViewer. C) Citation network, created in Litmaps, representing most connected papers at the top, and most recent papers on the right. Bubble size reflects relative citation number, and arrows point to articles cited. [file 12915_2026_2556_MOESM6_ESM.png]
